# Determination of a Predictive Cleavage Motif for Eluted Major Histocompatibility Complex Class II Ligands

**DOI:** 10.3389/fimmu.2018.01795

**Published:** 2018-08-06

**Authors:** Sinu Paul, Edita Karosiene, Sandeep Kumar Dhanda, Vanessa Jurtz, Lindy Edwards, Morten Nielsen, Alessandro Sette, Bjoern Peters

**Affiliations:** ^1^Division of Vaccine Discovery, La Jolla Institute for Allergy and Immunology, La Jolla, CA, United States; ^2^Department of Bio and Health Informatics, Technical University of Denmark, Lyngby, Denmark; ^3^Instituto de Investigaciones Biotecnológicas, Universidad Nacional de San Martín, San Martín, Argentina; ^4^Department of Medicine, University of California San Diego, La Jolla, CA, United States

**Keywords:** antigen processing, ligand elution, epitope prediction, human leukocyte antigen, major histocompatibility complex class II, CD4^+^ T cell epitopes, natural cleavage motif

## Abstract

CD4^+^ T cells have a major role in regulating immune responses. They are activated by recognition of peptides mostly generated from exogenous antigens through the major histocompatibility complex (MHC) class II pathway. Identification of epitopes is important and computational prediction of epitopes is used widely to save time and resources. Although there are algorithms to predict binding affinity of peptides to MHC II molecules, no accurate methods exist to predict which ligands are generated as a result of natural antigen processing. We utilized a dataset of around 14,000 naturally processed ligands identified by mass spectrometry of peptides eluted from MHC class II expressing cells to investigate the existence of sequence signatures potentially related to the cleavage mechanisms that liberate the presented peptides from their source antigens. This analysis revealed preferred amino acids surrounding both N- and C-terminuses of ligands, indicating sequence-specific cleavage preferences. We used these cleavage motifs to develop a method for predicting naturally processed MHC II ligands, and validated that it had predictive power to identify ligands from independent studies. We further confirmed that prediction of ligands based on cleavage motifs could be combined with predictions of MHC binding, and that the combined prediction had superior performance. However, when attempting to predict CD4^+^ T cell epitopes, either alone or in combination with MHC binding predictions, predictions based on the cleavage motifs did not show predictive power. Given that peptides identified as epitopes based on CD4^+^ T cell reactivity typically do not have well-defined termini, it is possible that motifs are present but outside of the mapped epitope. Our attempts to take that into account computationally did not show any sign of an increased presence of cleavage motifs around well-characterized CD4^+^ T cell epitopes. While it is possible that our attempts to translate the cleavage motifs in MHC II ligand elution data into T cell epitope predictions were suboptimal, other possible explanations are that the cleavage signal is too diluted to be detected, or that elution data are enriched for ligands generated through an antigen processing and presentation pathway that is less frequently utilized for T cell epitopes.

## Introduction

Major histocompatibility complex (MHC) class I molecules are expressed in virtually all nucleated cells, and their main biological function is to present peptides derived from processing endogenous antigens to killer CD8^+^ T cells (1). By contrast, MHC class II molecules are primarily expressed by professional antigen-presenting cells (APCs) such as macrophages, dendritic cells, and B cells, and are mostly involved in binding and presentation of peptides generated from exogenous antigens to CD4^+^ T cells through the endocytic pathway of antigen presentation (2). MHC II molecules are also expressed by non-professional APCs such as endothelial cells, fibroblasts, epithelial cells, and tumor cells when induced by inflammatory signals (3). In the main endocytic pathway of antigen presentation, extracellular antigens are internalized by phagocytosis, macropinocytosis, or receptor-mediated endocytosis, and degraded in acidic and proteolytic compartments such as lysosomes or late endosomes by proteases generally called cathepsins (4). Less frequently, MHC II molecules also bind peptides generated from processing of endogenous antigens, namely cytosolic and nuclear proteins, acquired through autophagy (5).

The complexes of peptides generated from antigen processing bound to MHC II molecules are transported to the cell surface, where they become available for recognition by CD4^+^ T cells. CD4^+^ T cell recognition of MHC class II presented peptides plays a critical role in diverse immune reactions such as immunity against viral and bacterial infections and parasitic infestations, as well as those involved in allergic reactions. In addition to these well-established roles, it is also understood that MHC class II antigen presentation pathway is involved in autoimmunity (1, 6) and cancer immunity (7–9). This broad immunological function makes the identification of MHC class II restricted peptides recognized as CD4^+^ T cell epitopes an important research area.

For MHC class I restricted T cell epitopes, the identification of T cell epitopes is aided by MHC class I binding predictions, which drastically reduce the number of peptides that have to be tested, as the vast majority of T cell epitopes bind in the top 0.5–2% of predicted binding peptides (10). By contrast, for MHC class II restricted T cell epitopes, the binding predictions are not as reliable (11), and epitopes are often in the top 10 or 20% of predicted binders. This is only partly a result of the predicted ligand affinities for MHC class II being less reliable compared to that of MHC class I. MHC class II binding predictions have become continuously more reliable (12–16), but the ability to predict MHC class II T cell epitopes is still substantially worse than for MHC class I. This suggests that factors beyond MHC class II binding affinity contribute to likelihood of a peptide to be recognized by CD4^+^ T cells.

It is widely recognized that natural processing shapes which peptides are available for binding to MHC molecules and subsequently presented to T cells, and that the capacity to bind MHC molecule is a necessary but not sufficient requisite for immunogenicity. Several algorithms are available which can assist in the prediction of which peptides are natural class I ligands (17, 18). Natural ligands have also been recently used to train class I predictive algorithms (10, 19, 20) leading to an overall improved performance for ligands and CD8^+^ T cell epitopes. By contrast, no algorithm is currently available to predict which peptides are naturally processed by the MHC class II antigen-presenting pathway. It is reasonable to hypothesize that analysis of MHC class II antigen processing and the resulting natural ligands could result in improving the epitope prediction algorithms and cast additional light on the processing mechanism itself.

Recent advances in mass spectrometry (MS)-based techniques have led to the identification of large amounts of peptides eluted from MHC molecules (21–25). These naturally processed peptide sets, called human leukocyte antigen (HLA) ligandome or HLA peptidome, are a valuable resource in expanding current knowledge about mechanisms of antigen processing. In this study, we analyzed sets of naturally processed MHC class II ligands identified by MS of peptides eluted from MHC class II-expressing cells and downloaded from the immune epitope database (IEDB) (26) to investigate MHC class II antigen-processing mechanisms and examine whether this information could be used to improve prediction of the CD4^+^ T cell epitopes.

## Materials and Methods

### Collection and Screening of Ligand Data

Major histocompatibility complex class II ligand elution assay data was collected by querying the IEDB database^1^ (26) using the following criteria: “Positive Assays Only, Epitope Structure: Linear Sequence, No T cell assays, No B cell assays, MHC ligand assays: MHC ligand elution assay, MHC Restriction Type: Class II.” The collected data included ligand sequence, details of the source from which the ligands originated including the source protein name, position of the ligand in the source protein, source organism name, and the restricting allele of the ligand. A Python script was used to parse the exported data. The source sequences of the ligands were collected from UniProt.^2^

**Figure 1:**
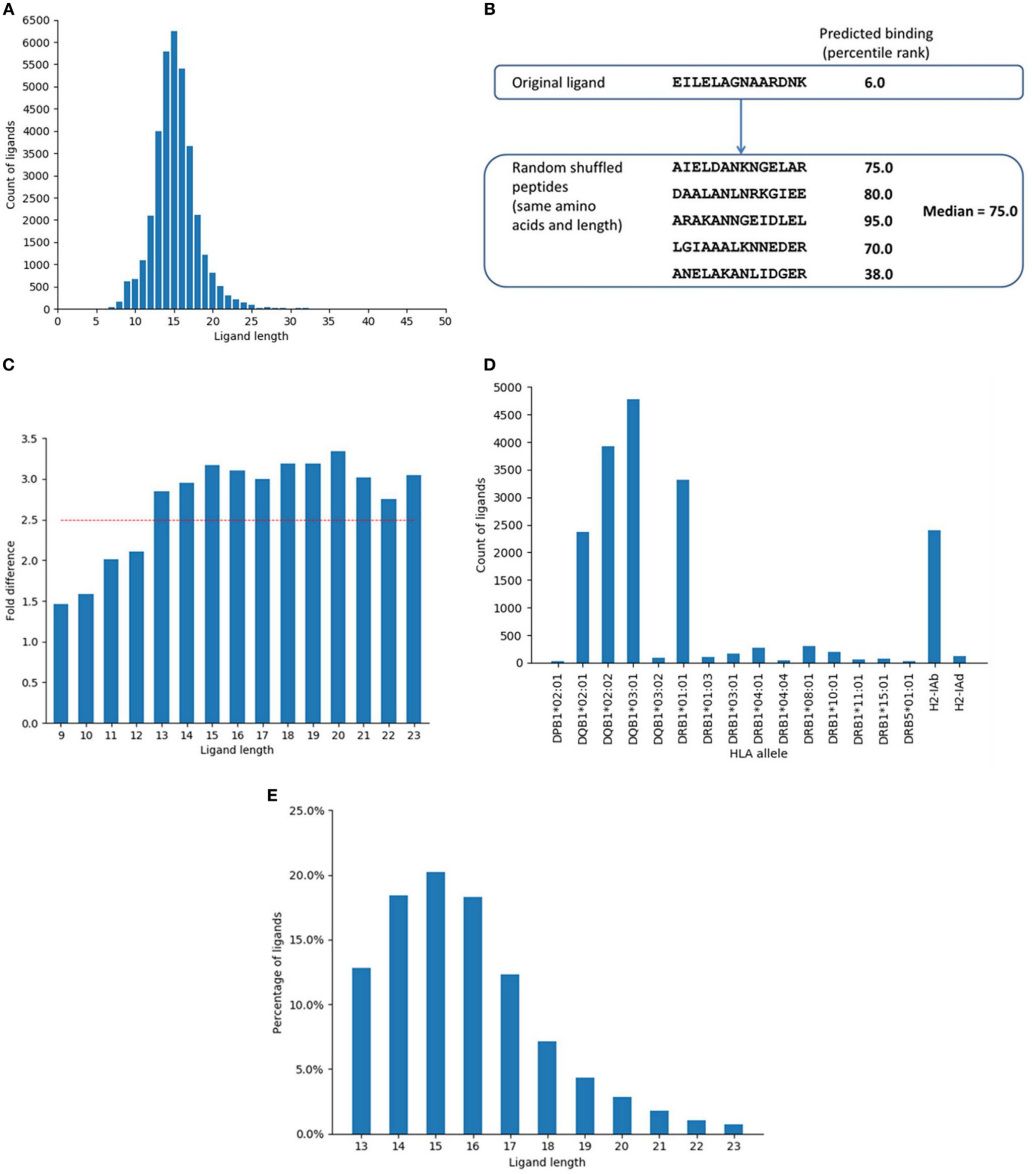
Selection of major histocompatibility complex (MHC) II ligand data and distribution of the data. **(A)** Distribution of ligand entries based on ligand length as collected from immune epitope database. The most abundant length was 15, followed by 14 and 16. Lengths 14–16 comprised around 50% and lengths 13–17 represented around 71% of the ligand data. **(B)** Example of random peptides generated by shuffling the amino acid residues from a ligand and corresponding predicted MHC II binding (percentile rank). Five random peptides were generated from each ligand by shuffling the component amino acids and the median of the predicted binding (percentile ranks) of the random peptides was assigned as the predicted percentile rank of the random peptide. **(C)** Fold difference between the proportion of predicted binders among ligands and shuffled peptides for different peptide lengths. The fold difference plateaued after reaching 2.5. The red line indicates fold difference 2.5. **(D)** Distribution of ligand data based on restricting alleles (only B-chain is shown). **(E)** Distribution of the ligand data based on final selected lengths.

Two independent sets of ligand data were used in the study. The first dataset was collected in July 2016 and contained 35,367 ligands reported in IEDB up to that point. The collected data were screened based on factors such as ligand length and allele distribution. The length of the ligands in the original dataset ranged from 3 to 46 and the distribution of ligands varied widely with respect to length (Figure 1A). The most abundant length was 15, followed by 14 and 16. These three lengths together comprised almost 50% of the data. From the initial dataset, first we selected ligands with lengths that represented at least 0.5% of the total ligands, which included lengths 9–23. This comprised 98.22% of the total data collected. Next, the restricting alleles of the ligands in the dataset were analyzed and entries with alleles listed unambiguously were selected. For example, some entries where the HLA alleles were listed as just the gene locus (HLA-DQ and HLA-DR), or alleles from chicken, horse, cow, and mouse for which we did not have binding prediction algorithms, were excluded. This was done to streamline the analyses. Representative alleles were assigned for entries where serotypes were indicated (e.g., DRB1*07:01 for HLA-DR7). Redundant entries with identical ligand sequence and alleles were removed. From this set, alleles with less than 50 entries were excluded. Some entries for which the source proteins were not clearly annotated were also excluded. We did further analyses to identify optimal ligand lengths as well as potential false-positive antigens (see Results). On the basis of these analyses, only ligands of lengths 13–23 were included, and ligands from 199 potential false-positive antigens were excluded (Table S1 in Supplementary Material). The final dataset contained 14,051 unique ligand entries that came from 2,604 source proteins. Details of the number of ligand entries in each filtering step is shown in Figure S1 in Supplementary Material. This dataset was used as the training ligand dataset for identifying the cleavage motif and generating the method for prediction of ligands.

A second set of eluted ligand data was collected from IEDB at a later stage of the study using same selection criteria as the previous ligand data and filtered to include only the studies from 2016 and 2017 and that were not present in the initial ligand dataset. The dataset was screened to eliminate redundancy by removing duplicate peptides and peptides with source protein information not provided unambiguously were also excluded. As above, ligands of length 13–23 were selected. Ligands that matched with any of the ligands in the training dataset with 100% identity as well as ligands that came from the source parent protein sequences in the training data were excluded. The final set contained 3,648 unique peptides that came from 1,144 unique protein sequences. No selection for specific restricting MHC molecules was done, as the data were only used for validation studies that did not require knowing the MHC restrictions. The source sequences were collected from UniProt. This dataset was used as the evaluation ligand dataset.

### Collection of CD4^+^ T Cell Epitope Datasets

In addition to the ligand data, we collected different sets of CD4^+^ T cell epitopes. First among them were sets of 15-mer epitopes from studies done in our lab that were shown to be recognized by T cells, and that were tested in a consistent format (referred as “in-house epitope set”). The peptides in these sets were tested for immune recognition in cohorts of 18–91 donors by ELISPOT assays for secretion of one of the following cytokines: IFN-g, IL-5, IL-10, or IL-17. The details of epitope sets including the corresponding references of each study are listed in Table S2 in Supplementary Material. The details of the identification of these epitopes are described in the peer-reviewed references listed in the table. In some cases, the epitope sets were selected based on interim analysis and do not exactly match the final epitope lists in the published reports. A brief description of the peptide selection protocols is as follows: for the epitope set from Timothy grass (TG) known allergens, previous studies identified 20 epitopes that accounted for 79.5% of the total response to a set of TG-derived pollen antigens (Phl p allergens) in TG allergic individuals (27). Since some of these were not 15-mers, longer peptides were “deconstructed” to derive 15-mers spanning those longer peptides. A total of 41 15-mers were derived. For the epitope set from novel Timothy grass allergens (NTGA), 19 peptides were described to encompass an NTGAp19 peptide pool, which were selected to encompass at least 40% of the total IL-5 response directed against all NTGA peptides screened (28). For the epitope set from house dust mite allergens, the peptide set included the 34 most dominant peptides cumulatively accounting for 90% of the total allergen-specific response detected in the screen (29). In analogy with what described for the TG set, longer regions were deconstructed into 15-mers, which yielded 52 peptides in total. For the epitope set from cockroach allergens, 71 most dominant epitopes were selected based on total SFC values of >1,000 (30). For TB epitope set, we selected 65 15-mer epitopes identified from the vaccine candidate antigens and previously known epitopes that captured 80% of the response (31, 32). In total, 248 epitopes were included in this set. The source sequences were collected from UniProt.

As a second set of epitopes, we used CD4^+^ T cell epitopes identified by tetramer mapping studies (referred as “tetramer set”). These peptides were collected from IEDB with the following selection criteria: “Positive assays only, Epitope structure: Linear sequence, T Cell assays: qualitative binding/multimer/tetramer (tetramer), No B cell assays, No MHC ligand assays, MHC restriction type: Class II, Host organism: Homo sapiens (human) (ID:9606, human).” The collected dataset was filtered for keeping only 15-mer epitopes for which a source antigen protein ID was available. The dataset contained 122 unique epitopes. The source sequences were collected from UniProt.

In addition to the above epitope sets, we identified five studies curated by IEDB that contained 15-mer peptides spanning six proteins that were tested for immunogenicity in the context of HLA class II restricted T cells. We collected the epitopes from these studies which included 73 unique epitopes from 6 distinct proteins that are completely independent from the other datasets (referred as “IEDB epitope set”) (33–37). More details regarding collection of this dataset are available in Ref. (38). The source sequences were again collected from UniProt.

### Calculation of “*Cleavage Probability Score*” for Prediction of Ligands

We first used the amino acid preferences around cleavage sites identified from the MHC ligand data to derive a “*motif score*” for N- and C-terminuses that can be applied to any sequence. This was done by first calculating the frequencies of the three amino acid residues at the N- and C-terminuses of the peptides (N−1, N0, and N+1 for positions one preceding N-terminus, N-terminus, and one after the N-terminus, respectively and correspondingly C−1, C0, and C+1 positions for C-terminus). These were then divided by the background frequencies of corresponding amino acids in the protein set overall, and these relative amino acid frequencies were log transformed to calculate log-odds scores. For a given triplet of amino acids, we can now assign a score of them being a C-terminal or N-terminal cleavage, by simply calculating the sum of log-odds scores:

$$\text{N }-\;\mathrm{motif}\;\mathrm{score}={\text{log}}_{10}(F_{\text{N}-1})+{\text{log}}_{10}(F_{\text{N}0})+{\text{log}}_{10}(F_{\text{N}+1})$$

$$\text{C }-\;\mathrm{motif}\;\mathrm{score}={\text{log}}_{10}(F_{\text{C}-1})+{\text{log}}_{10}(F_{\text{C}0})+{\text{log}}_{10}(F_{\text{C}+1})$$

where *F*_N−1_, *F*_N0_, and *F*_N+1_ are the relative frequencies of amino acids at one position preceding the N-terminus of the peptide, N-terminus, and one after the N-terminus, respectively and correspondingly for C-terminus.

Next, we calculated the probability of the given peptide to be ligand based on the peptide length and N-motif and C-motif scores. We used the relative proportion of the peptides of each length in the training ligand set as the probability based on length (called length probability) (Table S3 in Supplementary Material). For calculating the N-motif and C-motif probability, the entire set of peptides were divided into bins of approximately equal number of peptides separately for N- and C-motifs, based on their N-motif and C-motif scores, respectively. The N-motif and C-motif probability of the peptides was then calculated based on the number of ligands in the corresponding bin (Tables S4 and S5 in Supplementary Material). The “*cleavage probability score*” for each peptide was then derived by calculating the product of the length probability, N-motif probability, and C-motif probability. Figure S2 in Supplementary Material shows an illustration of deriving *cleavage probability score* for a peptide based on its length, N-motif, and C-motif.

## Results

### Generation of a High Quality MHC Ligand Elution Dataset

The training ligand dataset contained more than 14,000 naturally processed ligands identified by MS of peptides eluted from MHC class II-expressing cells. The dataset was collected from IEDB database by querying for MHC II ligand elution assays. The initial dataset contained 35,367 peptides and was screened for lengths and unambiguous allele restrictions as mentioned in Section “Materials and Methods.” After initial screening, the dataset contained 28,007 peptides of lengths 9–23. Within this set, the length distribution of some of the peptides reported as ligands was suspiciously short/long, raising concerns that some of them might be enriched for degradation products or other contaminants, rather than being derived from peptides truly bound to MHC. We thus set out to determine if there was a bias for peptides of certain lengths to better conform with MHC binding motifs than peptides of other lengths. This was done based on binomial probability distribution comparing the proportion of predicted binders among ligands and random peptides generated by shuffling the ligand residues for each ligand length. Five sets of random peptides were generated from the ligand sequences by shuffling the amino acid residues within the ligands (Figure 1B). Binding affinity was then predicted for the original ligands and random peptide sets for their corresponding alleles using NetMHCIIpan-3.1 (12). The median of the predicted percentile ranks of the five random sets was calculated and assigned as the binding affinity of the random peptides.

Based on a predicted binding affinity cutoff of percentile rank 10.0, the number of predicted binders among the original ligands and the random peptide sets were calculated. We found that the fold differences between the proportion of predicted binders among ligands and shuffled peptides for different lengths plateaued after reaching 2.5 (Figure 1C). The lengths with a minimum fold difference of 2.5 were thus selected, which included lengths 13–23 and contained 24,099 peptides. The shortest length of 13 corresponded to a 9-mer binding core and 2 flanking residues on both sides of the binding core, which is in concordance with previous experimental reports for the minimal length of a high affinity MHC ligand, which showed that binding affinity drops of drastically for peptides shorter than 12–13 residues (39, 40). Notably, there was no decrease in this fold difference for long sequences up until 23, supporting that such very long peptides and potentially longer ones are indeed very capable of binding to MHC class II molecules, which again aligns with previous reports that even full length proteins can bind when sufficiently denatured (41).

Major histocompatibility complex ligand elution assays can possibly pick up peptides that are not actually bound to the MHC molecule, but instead are derived from degradation products of contaminating proteins that get co-eluted with the MHC:peptide complex, or that are derived from MHC molecules themselves. If that is indeed the case, then certain proteins that are more likely to be co-eluted should be enriched as a source of peptides that do not possess the sequence motif necessary for binding to the MHC, which would identify their source proteins as potential false-positive antigens. The same protocol described above (using binomial probability distribution) was used to identify such potential false-positive antigens and exclude them from the final dataset. The analysis identified proteins that had significantly less number of predicted binders among ligands than expected of random peptides. The percentile rank cutoff for selecting predicted binders was 10.0. The analysis identified 199 proteins as potential false positives, which were excluded (Table S1 in Supplementary Material). Some of the top proteins in this list were MHC chains such as HLA-DQ alpha and beta chains, HLA-DP beta chain, HLA-DM alpha and beta chains, and beta-2-microglobulin. Proteins expressed in the endoplasmic reticulum and golgi network such as Trans-Golgi network integral membrane protein 2 and Endoplasmin were also in the list. After excluding these proteins and limiting ligands to length 13–23, the dataset contained 18,286 ligand entries. Figure 1D shows the distribution of the ligand data based on the restricting HLA alleles. The data were dominated by ligands restricted by DQ alleles. Around 61% of the ligands in the dataset were restricted by the four DQ alleles while the 10 DR alleles were associated with 25% of the data and 14% of the ligands were restricted by the two mouse alleles. Overall, there was a lack of data comprising DP alleles, with only one allele from the DP locus and were very few ligands in the dataset that associated with this allele.

As a final filtering step, we excluded all ligands that were not found with 100% identity in their source parent proteins. This final set included 14,051 ligand entries that came from 2,604 unique protein sequences. Details of the number of ligand entries in each filtering step is shown in Figure S1 in Supplementary Material. Figure 1E shows the final distribution of the data based on ligand length. As before, the most frequent length among the ligands in the final set was 15, followed by 14 and 16. Lengths 13–17 represented more than 80% of the final ligand dataset. Overall, this selection process provided us with a dataset of MHC class II ligands with well-defined source antigens, known restriction, and which removes multiple potential sources of contaminants.

### Amino Acid Composition of MHC Ligand Boundaries Reveals Putative Cleavage Motif

**Figure 2:**
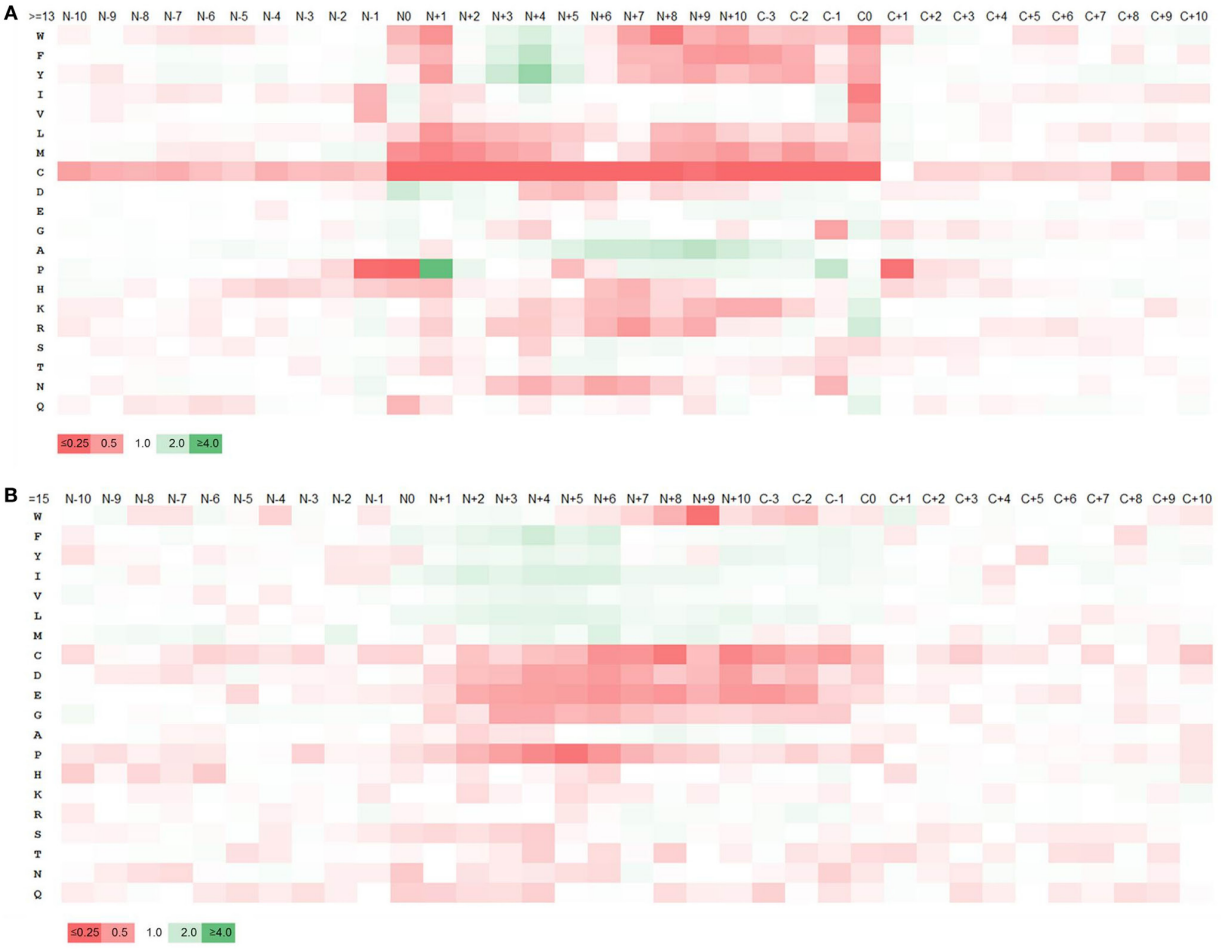
Enrichment and depletion of amino acids within and adjacent to major histocompatibility complex (MHC) II ligands and predicted MHC II binders. Heatmaps generated from the relative frequencies of amino acids at ligands/binders and nearby positions with respect to the overall amino acid frequency of the source proteins. “N” and “C” represents the N- and C-terminuses of the ligands, respectively and the numbers represent the amino acid positions with respect to N- and C-terminuses. The legend shows the color scale of the heat map with respect to the relative frequency of amino acids which is represented by the numbers on the legend. **(A)** Heatmap generated from ligands. **(B)** Heatmap from predicted binders. The pattern of amino acid enrichment and depletion was found to be significantly different between the heatmaps generated based on cleavage motif and binding motif.

Major histocompatibility complex ligands are cleaved from their source proteins by various enzymes. We hypothesized that the specificity of these enzymes would result in certain amino acids being favored over others at the cleavage sites at the start and end of MHC ligands. Accordingly, the amino acid composition of the ligands and adjacent regions was analyzed. The sequence regions analyzed included 10 residues prior and past the N- and C-terminuses of each ligand including residues at both termini. The frequency of the amino acids at each position was computed and the pattern of enrichment/depletion was analyzed by comparing it to the amino acid frequencies in the source proteins overall. As most ligands are ~15 residues in length, going in 10 amino acids from either terminus means that the enrichment/depletion motifs are expected to overlap, and in fact they do. Figure 2A shows the enrichment and depletion of amino acids at positions from the N-terminus ±10 positions; the C-terminal enrichment is shown from positions −3 to +10, because positions C−4 to C−10 from the C-terminus show essentially the same motif as the positions N+4 to N+10 from the N-terminus. We focused on the cleavage motif represented by the relative frequencies of amino acids at ligand termini and at the adjacent positions in comparison to the overall amino acid frequencies in the full length source proteins as shown in Table S6 in Supplementary Material.

The heatmap generated from the cleavage motifs showed prominent enrichment at N-terminus for P at position N+1 and D at N0, N+1, N+2 and at C-terminus for K and R at C0, I and V at C−1 and G, N and Q at C0 (Figure 2A). By contrast, the heatmap showed depletion of several hydrophobic residues, most prominently at the C0 and N+1 positions. We examined if the motif changed when we considered different ligand lengths by themselves, or only ligands restricted by HLA-DR or DP or DQ. We did not detect any significant differences; in fact, the motifs for all of the datasets we generated overlapped very closely (Tables S7 and S8 and Figures S3 and S4 in Supplementary Material). In all cases, the cleavage motifs at N- and C-terminuses were almost perfectly conserved, but the motif inside the ligands varied, which could be attributed to the difference in binding motif patterns associated with different alleles. This suggested that the motifs detected are not locus or peptide length dependent, but rather reflect general preferences for antigen processing, which is in line with the discovered motif matching to well-known cleavage motifs of proteases.

We also consistently detected a depletion of Cysteine residues not only within MHC ligands but also outside of them. While the depletion of Cysteines within eluted ligands could be due to experimental problems of these residues oxidizing and/or forming disulfide bonds, which would make them harder to detect, the fact that the Cysteine depletion is also present in flanking regions of the ligand suggests that, at least partially, Cysteine rich regions are poor for MHC ligand presentation, potentially due to their ability to form disulfide bonds that make them less accessible for MHC ligand generation (42–48).

To examine how the terminal sequence motifs discovered in our MHC ligand analysis compared to peptides that bind to MHC, whether they are naturally processed or not, we created a control dataset of peptides collected from the same source proteins of our ligand dataset, including only 15-mer peptides predicted to bind MHC using the 7-allele method as described in Ref. (49) based on the recommended universal threshold of 7-allele median consensus percentile rank 20.0 (for comparison, the average 7-allele median consensus percentile ranks of the 15-mer ligands was 33.57. There were ~38% 15-mer ligands below the threshold 20.0). An amino acid enrichment/depletion pattern was generated from this data the same way it was done for MHC ligands above, with the resulting heatmap shown in Figure 2B, and the numeric values reported in Table S9A in Supplementary Material. Overall, the motif obtained from peptides selected based on binding prediction was found to be distinct from that of the natural eluted MHC ligands. While there was overlap in the amino acid preferences at internal positions expected to be in contact with the MHC molecule, there was no sign of a cleavage motif at the termini for these predicted binders. For example, in contrast to natural ligand-based enrichment pattern, P and N were depleted at N-terminus cleavage positions and K and R were found to be depleted at the C-terminus. Similarly, enrichment of K and R at the C-terminus and depletion of C were less pronounced. We repeated this analysis using allele-specific binding predictions rather than the 7-allele methods, which led to identical results (Table S9B and Figure S5 in Supplementary Material). This confirms that the sequence motifs surrounding MHC ligand termini are due to processes other than MHC binding, such as cleavage of the ligands from their source protein.

### Prediction of MHC II Ligands Using the Terminal Cleavage Motifs

We wanted to determine if we could use the discovered cleavage motifs at the ligand termini in a predictive fashion. In a first analysis, we examined how well the motifs could re-identify the ligands in the training ligand data. The complete source sequences were broken down to all possible peptides of lengths 13–23. Peptides identical to the ligands were considered positive and all others were considered negative. The *cleavage probability scores* were estimated for each peptide (details in Section “Materials and Methods”), with the peptides with higher scores being predicted to have better chance to be ligands. In brief, the *cleavage probability scores*—the probability of a peptide being a ligand—were calculated based on peptide length and the cleavage motifs at N- and C-terminuses comprising the three amino acid residues at the N- and C-terminuses. We plotted the receiver operating characteristic curve (ROC curve) and estimated the area under the ROC curve (AUC) on a per-protein basis. In brief, the ROC curve plots the sensitivity as a function of the false positive rate of a binary classifier as the classification threshold is varied and the AUC is used as measure of performance of the prediction method. An AUC of 0.5 indicates completely random prediction, and an AUC of 1.0 indicates perfect prediction. In our case, the average AUC was found to be 0.851 when the performance was evaluated on a per protein basis and the AUCs were averaged. This value constitutes an upper boundary of prediction performance using this scoring scheme, as the motif values are applied to the same dataset from which they were generated.

### Validation of “*Cleavage Probability Score*” Using Independent MHC II Ligand Dataset

To verify the ability of the cleavage motifs to predict eluted ligands, we used an independent ligand dataset collected from the IEDB references published between 2016 and 2017, and excluded any ligand and protein sequences that were included in the training dataset. This evaluation set contained 3,648 unique peptides that came from 1,144 unique sequences. We predicted the ligands in the evaluation ligand data using the *cleavage probability scores* derived based on the cleavage motifs that were identified. The average AUC was 0.767 when the evaluation was done on a per protein basis and the AUCs were averaged.

### Improved MHC II Ligand Prediction by Combining Cleavage Motifs and Binding Motifs

We wanted to see if combining the scoring based on cleavage motifs with MHC binding predictions would improve the overall ability to predict MHC II ligands. We thus combined the cleavage motif based on *cleavage probability scores* derived above with the previously established “7-allele method” which is used for prediction of binding with MHC II alleles (49). In brief, the 7-allele method predicts the binding affinity of a peptide with seven HLA-DR alleles and calculates the median predicted affinity, which serves as a proxy for the ability of a peptide to bind promiscuously to different HLA molecules. Peptides with lower median percentile rank value are considered better predicted epitope candidates. The two scores were then combined with different weights as follows:

Combined score=α×cleavage probability score+(1−α)×binding score

where *cleavage probability score* is the score for the peptide based on the cleavage motif as explained above and *binding score* is the median percentile rank for the same peptide from the 7-allele method representing the MHC binding affinity. We used the sequences with 15-mer ligands only since the 7-allele method for binding prediction was available for only 15-mer peptides. The *cleavage probability scores* were converted to percentile ranks to adjust to the same scale as *binding scores*. The conversion to percentile ranks was done for each peptide with reference to the peptides from within the corresponding protein as reference.

The value of α was varied from 0 to 1 with interval of 0.1. Thus, with α = 0, the *combined score* was totally derived from *binding score* and with α = 1, the *combined score* was totally attributed by the *cleavage probability score*. Overall, we saw improvement in prediction performance for alpha values between 0.0 and 1.0, when both scoring schemes contribute rather than when either of the scoring methods was used alone. The results from this analysis are summarized in Table 1. When we applied the combined scoring approach on the training ligand data, the highest performance achieved was average AUC = 0.779 (α = 0.7), which was a slight improvement from the *cleavage probability score* alone (average AUC = 0.769). For the evaluation ligand data the performance improved to average AUC = 0.728 (α = 0.5) compared to *cleavage probability score* alone (average AUC = 0.700) (*p*-value = 0.02, paired *t*-test). A smaller improvement to AUC = 0.722 was observed when using α value of 0.7 that was optimal in the training set.

**Table 1:** Performance improvement with combined prediction approach in terms of area under the ROC curve (AUC).

Alpha	Average AUC—training ligand data	Average AUC—evaluation ligand data
0	0.591	0.630
0.1	0.635	0.665
0.2	0.675	0.693
0.3	0.710	0.712
0.4	0.738	0.723
0.5	0.759	0.728
0.6	0.774	0.726
0.7	0.779	0.722
0.8	0.778	0.716
0.9	0.774	0.708
1	0.768	0.700

*Comparison of the prediction performance when only one scoring method is used (binding-based when α = 0 and cleavage motif-based when α = 1.0) with the combined scoring approach where both binding- and cleavage motif-based scoring schemes were combined together. The AUC was highest for the training data at alpha = 0.7 and highest for evaluation data at alpha = 0.5*.

### Cleavage Motifs Fail to Predict CD4^+^ T Cell Epitopes

As we were able to use cleavage motifs to predict MHC II ligands, we wanted to see if we could use the same approach to improve CD4^+^ T cell epitope prediction. To test the ability of the *cleavage probability score* to differentiate actual epitopes from other peptides, we collected three separate sets of epitopes: one containing 15-mer epitopes from studies done in our lab that were shown to be recognized by T cells (“in-house epitope set”), another containing 15-mer CD4^+^ T cell epitopes identified by tetramer mapping studies (“tetramer set”), and a third one containing 15-mer epitopes curated by IEDB from studies done on overlapping peptides in six antigens (“IEDB epitope set”) (details of the peptide sets are provided in Section “Materials and Methods”).

The T cell epitopes in two of our datasets were discovered based on screens of consecutive 15-mer peptides overlapping by 10 residues that span their source proteins (the third one being the tetramer dataset). Such overlapping peptide datasets avoid potential biases from pre-selection of peptides tested for T cell recognition based on their predicted ability to bind MHC. Importantly, the N- and C-terminal boundaries of these peptides are a result of this overlapping peptide synthesis scheme, and thus the epitope peptide termini are not expected to be efficient cleavage sites. This does not impede their recognition by epitope specific memory T cells, as the peptides are supplied liberated from their source sequence. As long as the tested peptide can bind the MHC molecule and contains the amino acid sequence that primed the naive T cell receptor (TCR), the memory TCR can recognize it. So, while the 15-mer peptides recognized by memory T cells in our dataset do not have to have efficient cleavage sites at their termini, there needs to be overlapping priming peptide sharing a ~9-mer core that engage the TCR, and that can be efficiently liberated from the source protein.

Taking these into consideration, we calculated a score for each 15-mer in a protein that evaluates the ability to cleave peptides overlapping with this 15-mer by at least nine residues. For this purpose, we first calculated probability scores for all peptides of lengths 13–23 for all sequences in each of the three epitope sets. Then, for each 15-mer peptide in the protein, we calculated the average score among the peptides that shared 9-mer binding core with it and assigned it to the given peptide. The 15-mer peptides that matched exactly with the epitopes were labeled positive, and 15-mers that shared less than nine residues with any of the epitopes were labeled as negative. We plotted the ROC curve on a per protein basis and the average AUC was calculated for the three datasets. We found that the AUC was around 0.5 for all three datasets, showing that the *cleavage probability scores* failed to identify the CD4^+^ T cell epitopes. Using the maximum score of any of the overlapping peptides for a given 15-mer instead of the average score also gave similar AUC values around 0.5, suggesting that we could not detect an increase in cleavage efficiencies of CD4^+^ T cell epitopes.

Furthermore, we wanted to see if combining the *cleavage probability scores* with predicted *binding scores* (predicted median percentile rank from the 7-allele method representing the MHC binding affinity) could improve the prediction performance. For this, we predicted the binding affinity of all 15-mers in terms of “median percentile rank” (here called *binding score*) using the “7-allele method.” Same as before, for each peptide we calculated the average *binding score* among the peptides that shared 9-mer binding core with it and this average *binding score* was assigned to the given peptide. We first used the predicted *binding scores* alone to predict the epitopes and the AUCs for the in-house epitope set, tetramer set, and IEDB epitope set were 0.649, 0.747, and 0.668, respectively. For combining these *binding scores* with the *cleavage probability scores*, we first used the same approach as before using α parameter with the value of α being varied from 0 to 1 with interval of 0.1. The *cleavage probability scores* were converted to percentile ranks to adjust to the same scale as *binding scores* as before. Thus, with α = 0, the *combined score* was totally contributed by *binding score* alone and with α = 1, the *cleavage probability score* attributed the complete *combined score*. But we found that any α > 0 did not improve the AUC. In all three datasets, the best performance was when *binding scores* were used alone.

Since a linear combination of cleavage motif prediction and binding prediction using α parameter did not show improvement we wanted to try an alternate approach. For this, we first classified the peptides into MHC “binding” and “non-binding” based on the recommended median percentile cutoff-score of 20.0. We calculated the AUC using this “binary” binding data and the AUCs for the in-house epitope set, tetramer set, and IEDB epitope set were 0.538, 0.596, and 0.582, respectively (average = 0.572). Furthermore, we replaced the *cleavage probability scores* of the predicted non-binders with “poor” score (=0.0) while the predicted binders retained the *cleavage probability scores* that we calculated (all of which are >0.0). We did prediction performance evaluation with the updated *cleavage probability scores* which contained the binding information and found the AUCs to be 0.537, 0.601, and 0.555, respectively for the above epitope sets (average = 0.564). Overall, the AUCs for the combined scoring scheme were not significantly better compared to the corresponding AUCs that we obtained when the predicted binding scores alone were used.

### Availability of the Method

An online tool for predicting the MHC class II ligands is made available at the IEDB Analysis Resource website at http://tools.iedb.org/mhciinp. The stand-alone and API versions of the tool will be made available in the future at the IEDB Analysis Resource website as well.^3^

## Discussion

Binding of peptides to MHC molecules is considered to be the limiting factor in the T cell epitope antigen processing and presentation pathway, but binding in itself is not sufficient to generate an immune response (50). Thus, most epitope prediction algorithms rely on MHC-peptide binding data for training algorithms. This provides reliable information regarding the “binding motifs” of peptides to MHC molecules, but lacks information on how the peptides are cleaved naturally from the antigens. Using data from more than 14,000 naturally processed peptides that were eluted from MHC II molecules, we uncovered a robust “cleavage motif” surrounding the N-terminal and C-terminal ends of naturally processed ligands within antigens. The cleavage motifs had various degrees of enrichment of P and D at N-terminus and K, R, I, V, G, N, and Q at C-terminus. At the same time several other residues were depleted at N- and C-terminuses, mostly the hydrophobic residues. The residues W, F, Y, I, V, L, M, and C were depleted at various degrees at different positions at both N- and C-terminuses. P was also depleted at N−1, N0, and C+1 positions. This cleavage motif information was used to generate a scoring scheme which was in turn used in devising a new method for predicting MHC II ligands. This cleavage motif is universal to MHC class II ligands, independent of the restricting allele.

As mentioned above, cleavage motifs had various degrees of enrichment of P and D at N-terminus and K, R, I, V, G, N, and Q at C-terminus. This gives insight on the specificity of the processing itself. First of all, it has long been recognized that processing can occur as a result of several types of proteases (51), including a prominent role for cathepsins (52). Indeed, the enrichment for D at the N-terminus is consistent with the reported role of aspartic proteases cathepsins D and E (53). The enrichment for P is consistent with the fact that its α-amino group is secondary rather than primary as other amino acid, and is therefore unavailable for degradation from aminopeptidases. Furthermore, the K/R C-terminal enrichment is the hallmark of a trypsin-like specificity. Trypsin was in fact used to define the first class II restricted epitope in history, back in the early 1980s (54).

In addition to the enrichment of residues that are consistent with cleavage of peptide termini by proteases, several hydrophobic amino acids were found to be depleted at various positions at N- and C-terminuses, and also Cysteine residues were depleted throughout the vicinity of the ligands. This could be due to factors other than cleavage motifs that influence antigen processing and presentation (55). Specifically, the antigen three-dimensional structure can influence proteolytic processing and therefore govern the presentation of individual ligands and determine the rate of efficiently presenting peptides to T-cells, which would be the case for the hydrophobic core of proteins, or residues between disulfide bonds formed by Cysteine pairs (56–59).

Moreover, we cannot exclude that the use of MS for identification of the naturally eluted peptides contributes for some of the detected motifs. It is expected that, for example, the presence of charged amino acids impacts the ability to detect peptides. However, it is not expected that such favored amino acids is concentrated at the ends of the peptides, and there should definitely be no influence of residues flanking the eluted ligand in its source protein. This makes us confident that the major contributor to the detected motif in the residues immediately surrounding naturally processed ligands is reflecting cleavage preferences.

The comparison of MHC class I and class II restricted ligands is informative. For MHC class I, it has long been shown that the processing machinery starting with proteasomal cleavage, TAP transport, and trimming in the ER shapes the ligand repertoire available for binding to MHC class I molecules. However, the predominant position in a peptide that determines its ability to bind to MHC class I molecules is the C-terminal residue, and that same residue predominantly determines its ability to be cleaved by the proteasome and transported by TAP. Presumably due to coevolution, both processes favor the same kind of amino acid residues, which makes sense as MHC molecules that bind peptides that they are not supplied with would not be efficient in displaying peptides to T cells (60, 61). Due to this overlap in peptide residues responsible for MHC class I binding and processing, combining predictions of the two has a comparably minor effect. By contrast, the MHC class II ligand cleavage motif we describe here is flanking the residues responsible for peptide binding to MHC class II molecules, and does not overlap with the binding motif. This explains why combining MHC class II binding and cleavage predictions provide substantial improvements to identify the ability to predict MHC class II ligands.

This study incorporates cleavage and binding motifs into prediction of MHC II ligands but it is important to note that several other factors could also contribute in ultimately determining the epitope repertoire and immunogenicity. For example, HLA-DM, which interacts with MHC II–class II-associated invariant chain peptide (CLIP) complexes and dissociates CLIP from MHC binding groove, is believed to influence CD4^+^ T cell epitope repertoire (4, 62). The relative surface accessibility of different regions of the antigens, mostly determined by the structure of the antigens, may also be a contributing factor to the overall epitope repertoire (48). Composition of the amino acids that come in contact with TCR has been shown to influence the immunogenicity in MHC class I antigen presentation (63) and same could be true in case of MHC class II as well. Incorporation of these factors as well as additional parameters such as recognition of epitopes by sets of known TCRs could potentially narrow this down further (64).

We want to stress that the cleavage motif described here, and the prediction method derived, are intentionally kept very simple. We simply calculated ratios of amino acid frequencies to obtain the C-terminal and N-terminal cleavage motifs. No fitting of our combined MHC binding and processing model to T cell epitope data was performed apart from varying a single scaling parameter (alpha) that combines the two scores. We fully expect that more complex machine learning approaches will be able to further improve on the performance reported here. The purpose of keeping things simple is to demonstrate for the first time that the MHC class II binding motif and the cleavage motif independently contribute to the likelihood of a peptide being an MHC II ligand.

Despite the ability of the cleavage motifs to predict the MHC II ligands, it failed to predict the CD4^+^ T-cell epitopes in our epitope sets, both alone and in combination with MHC binding predictions. As discussed before, there could be several other factors that influence antigen presentation and recognition by TCRs. Given that peptides identified as epitopes based on CD4^+^ T cell reactivity typically do not have well-defined termini, it is possible that motifs are present but outside of the mapped epitope. Our attempts to take that into account computationally did not show any sign of an increased presence of cleavage motifs around well-characterized CD4^+^ T cell epitopes. It could also be possible that the cleavage precursor signals in the ligands are getting diluted in the epitope data and are unable to be recovered this way. Besides, since we are attempting to use the cleavage information in an “allele-agnostic” way, i.e., not considering the HLA allele restriction of the peptides, the approach may be unable to capture MHC-specific signals. While it is possible that our attempts to translate the cleavage motifs in MHC class II ligand elution data into T cell epitope predictions were suboptimal, another possible explanation is that such elution data are enriched for ligands generated through an antigen processing and presentation pathway that is less frequently utilized for T cell epitopes.

## Author Contributions

BP conceived and supervised the project. SP, EK, SD, VJ, and LE compiled the data. SP did the analysis. BP, AS, and MN reviewed the results. SP, BP, and AS wrote the manuscript. All authors reviewed, edited, and approved the final manuscript.

## Conflict of Interest Statement

The authors declare that the research was conducted in the absence of any commercial or financial relationships that could be construed as a potential conflict of interest.

## Footnotes

^1^www.iedb.org (Accessed: 2016–2018).

^2^www.uniprot.org (Accessed: 2016–2018).

^3^http://tools.iedb.org/ (Accessed: 2016–2018).

## Supplementary Material

Figure S1 Details of the number of ligand entries in the training data in each filtering step.

Figure S2 Illustration of deriving *cleavage probability score* for a peptide based on its length, N-motif, and C-motif.

Figure S3 Heatmaps showing the pattern of relative enrichment/depletion of amino acids at ligand and nearby 10 positions, generated from ligands of each length separately. Panels **(A–K)** represent each of the lengths considered 13–23, respectively.

Figure S4 Heatmaps showing the pattern of relative enrichment/depletion of amino acids at ligand and nearby 10 positions, generated from ligands excluding different loci. Ligands of all lengths are considered (13–23). Panels **(A–C)** represent datasets with no DQ alleles, no DR alleles, and no mouse alleles, respectively.

Figure S5 Heatmap showing the pattern of relative enrichment/depletion of amino acids at ligand and nearby 10 positions, generated from 15-mer peptides from source sequences selected based on binding affinity prediction, when threshold for individual allele was used (immune epitope database consensus percentile rank 10.0).

## Abbreviations

APCs, antigen-presenting cells; AUC, area under the ROC curve; CLIP, class II-associated invariant chain peptide; FPR, false positive rate; HLA, human leukocyte antigen; IEDB, immune epitope database; MHC, major histocompatibility complex; MS, mass spectrometry; ROC curve, receiver operating characteristic curve; TCR, T cell receptor.
