# Supplementary figures and images for: Determination of a Predictive Cleavage Motif for Eluted Major Histocompatibility Complex Class II Ligands
Source: Front Immunol. 2018 Aug 6;9:1795. doi: 10.3389/fimmu.2018.01795 (PMC6087742; doi:10.3389/fimmu.2018.01795)

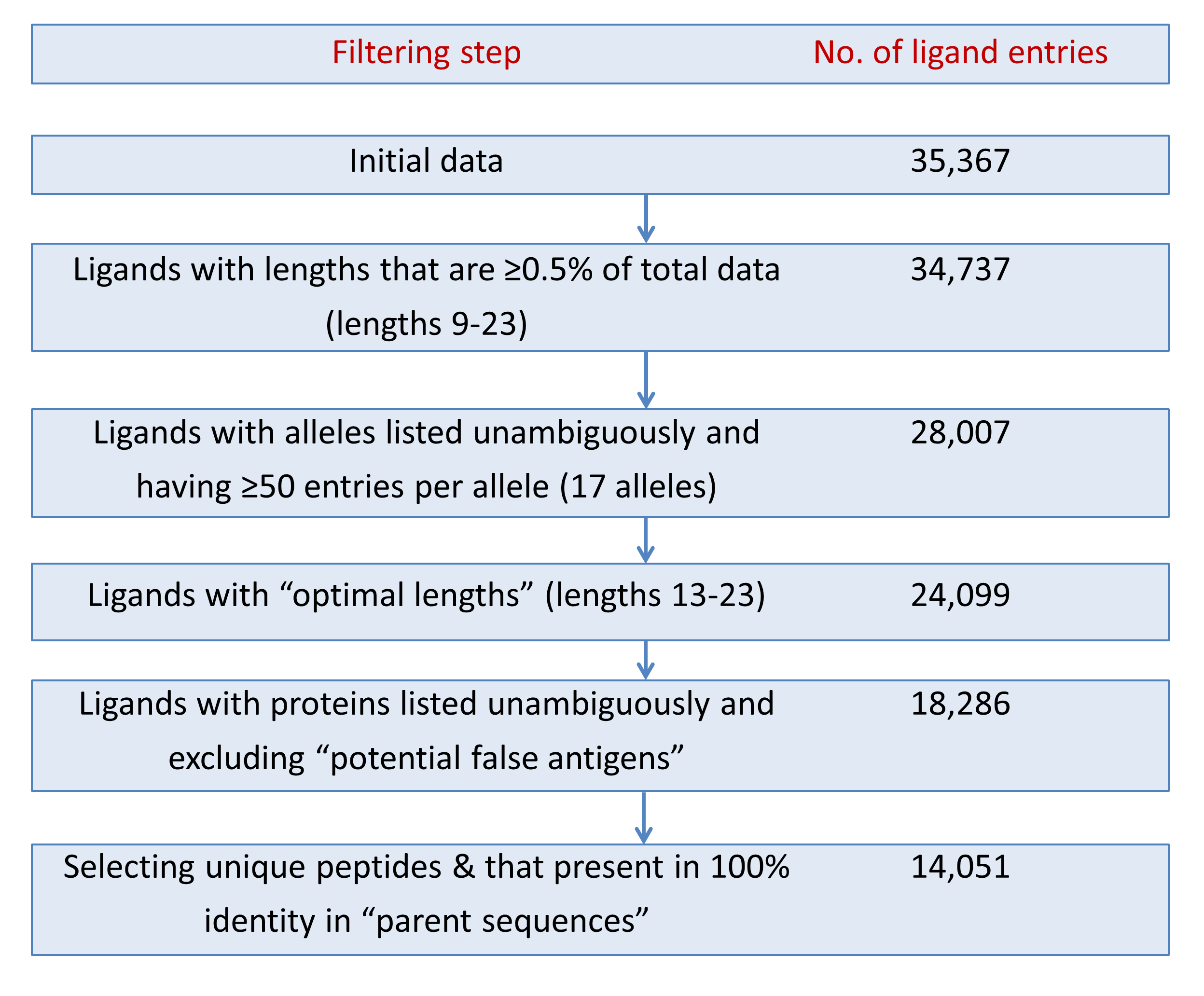

Supplement: Figure S1 — Details of the number of ligand entries in the training data in each filtering step. [file image_1.jpg]

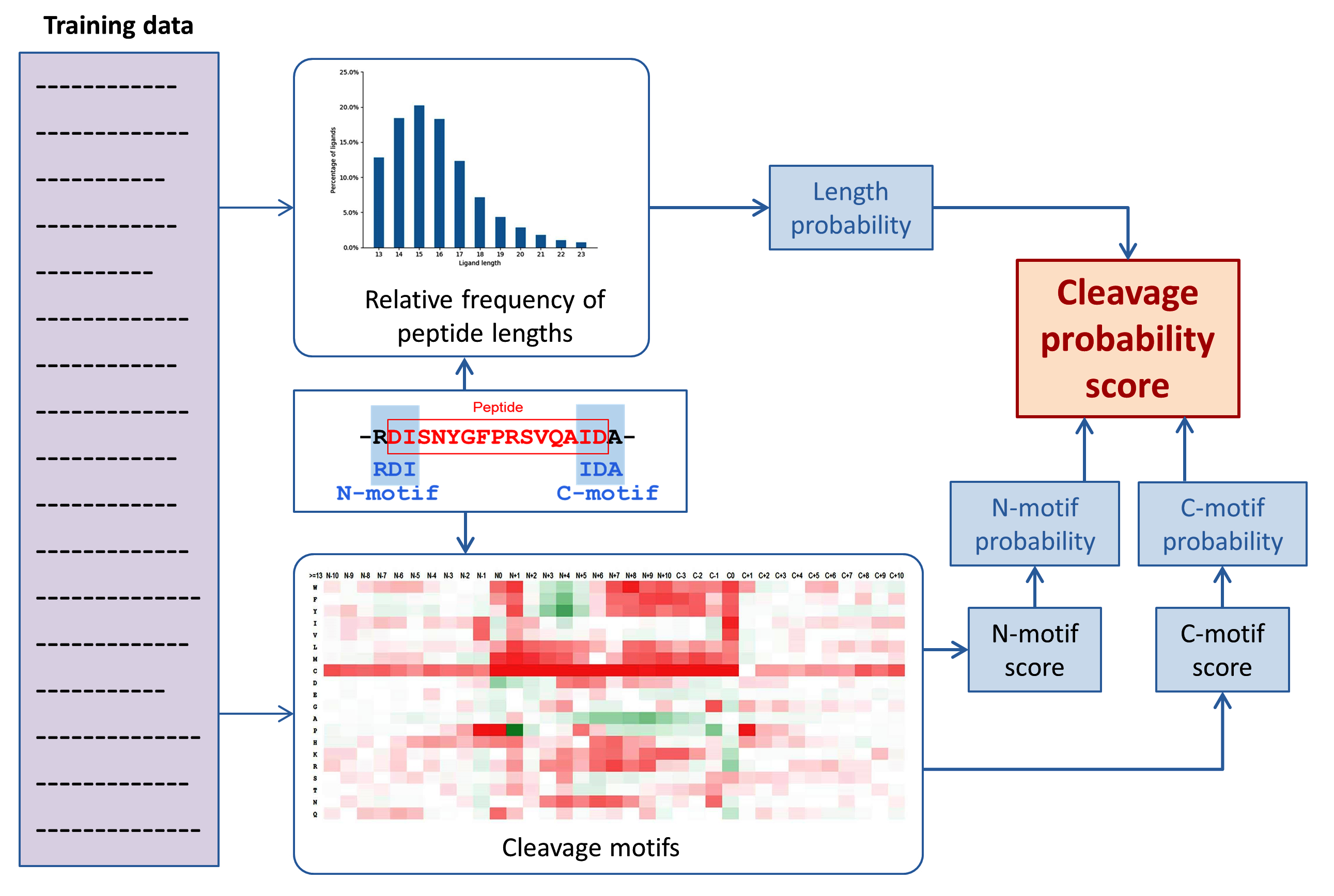

Supplement: Figure S2 — Illustration of deriving cleavage probability score for a peptide based on its length, N-motif, and C-motif. [file image_2.jpg]

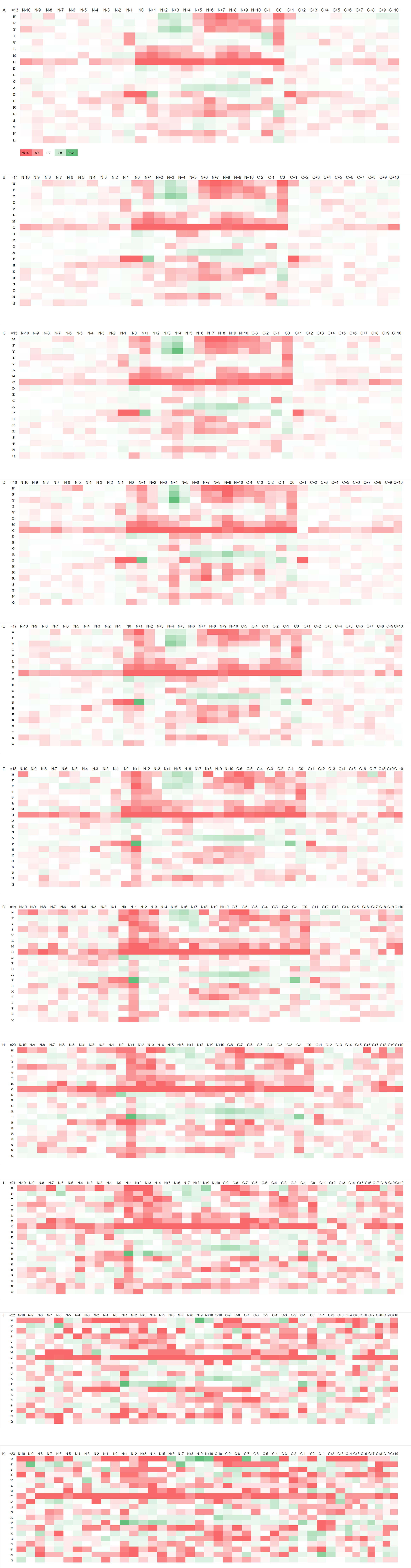

Supplement: Figure S3 — Heatmaps showing the pattern of relative enrichment/depletion of amino acids at ligand and nearby 10 positions, generated from ligands of each length separately. Panels (A–K) represent each of the lengths considered 13–23, respectively. [file image_3.jpg]

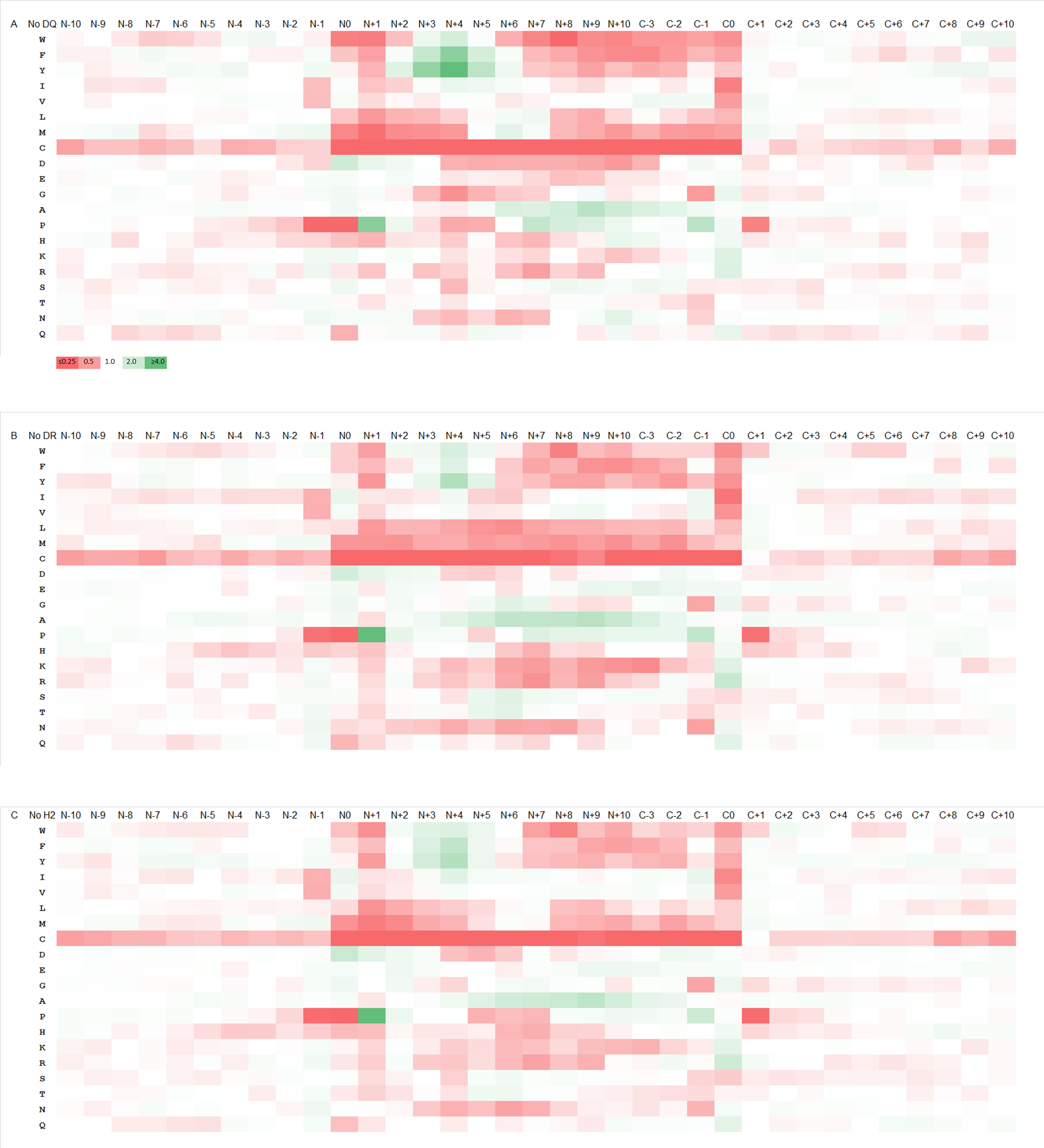

Supplement: Figure S4 — Heatmaps showing the pattern of relative enrichment/depletion of amino acids at ligand and nearby 10 positions, generated from ligands excluding different loci. Ligands of all lengths are considered (13–23). Panels (A–C) represent datasets with no DQ alleles, no DR alleles, and no mouse alleles, respectively. [file image_4.jpg]

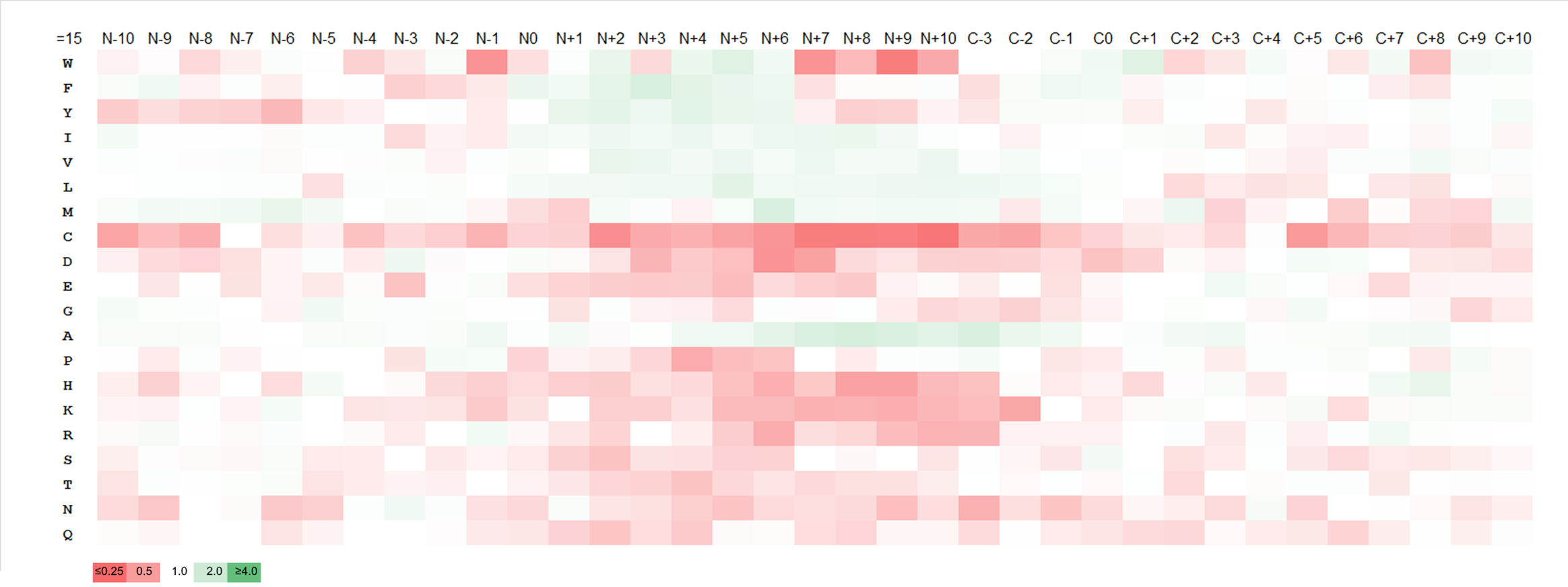

Supplement: Figure S5 — Heatmap showing the pattern of relative enrichment/depletion of amino acids at ligand and nearby 10 positions, generated from 15-mer peptides from source sequences selected based on binding affinity prediction, when threshold for individual allele was used (immune epitope database consensus percentile rank 10.0). [file image_5.jpg]
